# Should carbohydrate-modified diets be the first option for weight loss in people with impaired glucose metabolism? A scoping review

**DOI:** 10.1111/obr.13706

**Published:** 2024-02-14

**Authors:** Katie M. Ellison, Holly R. Wyatt, James O. Hill, R. Drew Sayer

**Affiliations:** 1 Department of Nutrition Sciences, The University of Alabama at Birmingham, Birmingham, Alabama, USA; 2 Department of Family and Community Medicine, The University of Alabama at Birmingham, Birmingham, Alabama, USA

**Keywords:** carbohydrate-modified diets, impaired glucose metabolism, precision nutrition, weight loss

## Abstract

While the “precision nutrition” movement is at an early stage of development, several investigations have compared low-fat versus carbohydrate (CHO)-modified diets (i.e., low-or-reduced-CHO, low glycemic index/load diets, and high-fiber) in people with normal versus impaired glucose metabolism. The purpose of this scoping review was to summarize evidence in support of the hypothesis that CHO-modified diets are more effective for weight loss among people with impaired glucose metabolism. Fifteen articles were included in this review: seven retrospective analyses of randomized clinical trials and eight prospective randomized clinical trials with prespecified hypotheses related to a diet (low-fat vs. CHO-modified) × phenotype (normal vs. impaired) interaction. Evidence in support of the hypothesis was identified in six of seven retrospective and three of eight prospective studies, which led to a recommendation of CHO-modified diets as a first-line option for people with impaired glucose metabolism. However, the evidence in support of this recommendation is relatively weak, and dietary prescriptions should consider additional contextual information that may influence overall dietary adherence. Additional and rigorous research using innovative randomized experimental approaches is needed for stronger dietary weight loss recommendations based on pretreatment glycemic status.

## 1 | INTRODUCTION

The persistently high prevalence of obesity has led to rates of type 2 diabetes (T2D), cardiovascular disease, and other obesity-associated chronic diseases reaching historic levels.^1^ Public health officials have long called for action on obesity prevention and treatment, but little progress has been made, especially with regard to lifestyle-based approaches such as diet and exercise. Decades of research have attempted to identify the best diet for sustainable weight loss, but the vast majority of people experience significant weight regain.^2^ There is much debate regarding the etiology of obesity and the optimum macronutrient distribution for weight loss—especially comparisons of reduced- or low-carbohydrate (CHO) and low-fat diets.^3–8^ Despite this disagreement, the scientific community seems to have reached one paramount conclusion: there is no one-size-fits-all approach for weight loss. This is reinforced by the considerable heterogeneity observed in the development and expression of obesity.^9^ It is reasonable to assume then that treatments for obesity should be similarly diverse. This has shifted attention toward identifying biological, behavioral, environmental, and psychosocial factors that may be used to predict an individual’s response to a weight loss intervention.^10^ Doing so would shift the debate of which dietary pattern is “most effective” to which dietary pattern is “most effective for whom.” Such a shift would prioritize allocating resources toward acquiring an evidence base for personalizing weight loss regimens based on individual and contextual factors affecting intervention response. This concept has been coined *precision nutrition* and stands to fundamentally alter the way nutrition research is conducted and how obesity is clinically treated.

For example, it has been suggested that the effectiveness of interventions to treat obesity may differ between individuals with normoglycemia compared with prediabetes or T2D as well as among the different phenotypes of prediabetes.^11^ Prediabetes is an umbrella term that describes multiple pathophysiologies of insulin resistance and insulin secretion that contribute to unique patterns of fasting and/or postprandial hyperglycemia.^12^ Three distinct phenotypes of prediabetes have been identified: impaired fasting glucose (IFG), impaired glucose tolerance (IGT), and a combination of IFG + IGT.^12,13^ Isolated IFG is defined as a fasting plasma glucose concentration between 5.6 and 7.0 mmol/L and results primarily from hepatic insulin resistance. When subjected to a standard 75-g oral glucose tolerance test (OGTT), 2-h plasma glucose concentrations will return to preload levels in individuals with isolated IFG because they maintain normal skeletal muscle insulin sensitivity and late-stage insulin secretory response (60–120 min). Conversely, isolated IGT principally results from skeletal muscle insulin resistance and is characterized by a normal fasting glucose concentration (<5.6 mmol/L) but an elevated 2-h glucose concentration (7.8–11.0 mmol/L) following an OGTT. People with IFG + IGT present with both hepatic and skeletal muscle insulin resistance. The characteristics of normal glucose tolerance (NGT), isolated IFG, isolated IGT, and IFG + IGT in response to an OGTT are summarized in Table 1. This detailed explanation of the subtypes of impairments in glucose metabolism underscores the complexity of conceiving, developing, and evaluating diet intervention strategies that are tailored to people with “impaired glucose metabolism,” a term that often conflates these unique conditions. The heterogeneity in the metabolic impairment(s) may very well require intervention approaches that are similarly unique as the underlying pathophysiology.

Given that dietary CHO are the primary signal for insulin secretion and that insulin inhibits lipolysis and promotes fat deposition, it is reasonable to hypothesize that pretreatment glycemic and/or insulinemic status may moderate weight loss responses to CHO-modified diets. This relationship between CHO intake and insulin action is the foundation for the Carbohydrate-Insulin Model of Obesity^14^ which has been the subject of many—sometimes contentious—scientific debates.^3–8^ Reducing dietary CHO has also been a consistently popular weight loss strategy touted in lay publications.^15^ Evidence supporting^16–19^ and refuting^20,21^ the Carbohydrate-Insulin Model abound in the scientific literature; vocal proponents and skeptics lead seemingly entrenched camps on both sides of the low-CHO versus low-fat diet debate. Meta-analytical evidence has consistently demonstrated that compared with low-fat diets, low-CHO diets produce marginally greater weight loss in the short- to intermediate-term (i.e., ≤ 6 months), but that these differences are not often maintained with longer duration follow-up. Importantly for the topic of this paper, the weight loss benefit of low-CHO diets has been demonstrated in people with^22,23^ and without T2D^24,25^ and may suggest that greater short-term weight loss may be achieved with a low-CHO diet, irrespective of glycemic status. However, a growing body of literature—summarized in this review— has begun to address whether pretreatment glycemic and/or insulinemic status may moderate weight loss success with low-CHO versus low-fat diets. In general, the most common hypothesis among known comparisons is that a low-CHO diet is superior for weight loss in people with impaired glucose or insulin metabolism, consistent with the Carbohydrate-Insulin Model.

Understanding the relationship between pretreatment status and the underlying mechanisms of weight loss with these interventions has the potential to accelerate the translation of personalized weight loss regimens to clinical and community settings and move the precision nutrition agenda forward. Therefore, the aim of this scoping review is to describe the current state of knowledge on the effects of pretreatment glycemic and/or insulinemic status on weight loss and weight loss maintenance (WLM) regimens prescribing varying amounts and types of dietary CHO. These dietary approaches will collectively be referred to as CHO-modified diets and include reduced-CHO (i.e., non-ketogenic) diets, ketogenic diets, low glycemic index/load diets, and high-fiber diets. Later sections of this review will summarize the existing literature on the utility of fasting plasma glucose (FPG), fasting insulin (FI), insulin sensitivity, and/or early-phase insulin secretory response (insulin concentration 30 minutes into an OGTT; INS-30) as predictors of weight loss or WLM with CHO-modified diets. As acknowledged above, these are unique physiological manifestations of “impaired glucose metabolism,” but the summary and conclusion of this review will refer to them broadly as impaired glucose metabolism. Studies included in the review will be categorized as retrospective secondary analyses (i.e., were not explicitly designed to test the effect of pretreatment glycemic and/or insulinemic status on weight loss) or prospective trials with a priori hypotheses related to effect modification of weight loss response by glycemic and/or insulinemic status. Finally, suggestions for continued research priorities in these areas are recommended.

## 2 | METHODS

### 2.1 | Search strategy

A university librarian was consulted regarding appropriate search strategies in the electronic databases PubMed, Web of Science, EMBASE, and ERIC. The following keyword combinations were searched using the AND/OR function: “pretreatment/baseline” AND “glucose/glycemia” OR “insulin/insulinemia,” as well as “weight loss/weight change” AND “carbohydrates/fiber/sugar.” Additional studies were also identified from the reference lists of identified articles. Studies were then screened for inclusion and exclusion criteria and organized by type, as defined below.

### 2.2 | Inclusion and exclusion criteria

Original peer-reviewed research studies were included if weight change was analyzed based on defined differences in pretreatment glycemic and/or insulinemic characteristics and compared weight loss between CHO-modified (i.e., reduced overall CHO intake, reduced glycemic index or load, or increased fiber) and comparator diet(s) with higher CHO, glycemic index/load intake, or lower fiber intake. Inclusion was limited to two study types: prospective randomized clinical trials with predefined analyses related to interactions of pretreatment glycemic and/or insulinemic status by diet type interactions for weight loss, and retrospective secondary analyses of randomized trials investigating these interactions. Studies were excluded if they were observational studies and analyses (prospective or retrospective) of non-randomized trials or did not clearly define dietary prescriptions or report weight loss outcomes. A total of 15 studies (seven retrospective and eight prospective) from 14 publications^26–39^ met the inclusion criteria for this review. Retrospective analyses of three randomized clinical trials (Diet, Obesity, and Genes [DiOGenes], Supermarket Intervention [SHOPUS], and Nutrient-Gene Interactions in Human Obesity [NUGENOB]) were conducted by Hjorth et al. to investigate whether pretreatment FPG and FI modified weight loss success in those trials.^26^ Each study will be described individually below, but the secondary analyses of these three trials were published in a single manuscript.^26^ In a prospective study, weight loss^32^ and WLM^33^ outcomes were reported in separate manuscripts, but these results are described in a single study in this review. A small prospective pilot study^38^ was conducted prior to the Diet Intervention Examining The Factors Interacting with Treatment Success (DIETFITS) trial,^39^ but the results of the pilot and main study are described in the same section below. Lastly, two secondary analyses of the DIETFITS trial are included in the section for that study to provide additional contextual information related to the study findings, but these are not formally included in the scoping review. Included retrospective and prospective studies including the specific CHO modification strategies, indicators of impaired glucose metabolism, and brief summary of significant results are included in Tables 2 and 3, respectively.

## 3 | EFFECT OF PRETREATMENT GLYCEMIC AND/OR INSULINEMIC STATUS ON WEIGHT CHANGE WITH CHO-MODIFIED DIETS

### 3.1 | Retrospective secondary analyses

#### 3.1.1 | DiOGenes study

In the DiOGenes study, 316 adults with overweight or obesity across eight European countries were randomized to either ad libitum high or low glycemic load diets after achieving at least 8% weight loss.^40,41^ A secondary analysis of 266 participants revealed that participants with prediabetes regained 5.83 kg (*P* < 0.001) more on the high glycemic load diet compared with the low glycemic load diet after 26 weeks. Conversely, those with normoglycemia at baseline (FPG < 5.6 mmol/L) only regained 1.4 kg more on the high glycemic load diet than on the low glycemic load diet (*P* = 0.003).^26^ When the DiOGenes participants were retrospectively stratified by FI, those with low FI regained 2.27 kg more on the high glycemic load diet compared with the low GL diet (*P* < 0.001), whereas no difference in weight regain was observed in those with high FI (*P* = 0.24).^26^ When considering both FPG and FI status, participants with prediabetes and low FI consuming a high glycemic load diet regained 7.78 kg more than those consuming the low glycemic load diet.^26^

#### 3.1.2 | SHOPUS trial

For the SHOPUS trial, 181 participants with central obesity were randomized to receive a high-fiber diet or a control diet lower in fiber and higher in fat for 26 weeks.^42^ A secondary analysis of 176 participants revealed that those with prediabetes lost an average of 6.04 kg more (*P* < 0.001) on the high-fiber diet than the control diet.^26^ Overall, a significant 3.84 kg difference was found between diets by glycemic status (*P* = 0.001). When the SHOPUS participants were stratified by FI status, those with low FI lost 4.09 kg more (*P* < 0.001) on the high-fiber versus control diet, and participants with high FI lost 1.61 kg more (*P* = 0.02) on the high-fiber diet.^26^ Considering FPG and FI together, participants with prediabetes and low FI lost 6.27 kg more on the high-fiber diet compared with the control diet.^26^ Upon further analysis, the authors estimated every mmol/L increase in baseline FPG predicted a between-diet difference of 3.00 kg (*P* = 0.001) greater weight loss from the high-fiber diet.^43^ Also, for each pmol/L, lower baseline FI in individuals with normoglycemia predicted a 0.039 kg greater weight loss from the high-fiber diet (*P* < 0.001).^43^

#### 3.1.3 | NUGENOB study

In a secondary analysis of the NUGENOB study, 743 participants with normoglycemia, prediabetes, or T2D consumed either a calorie-restricted high-fat, low-CHO diet or a low-fat, high-CHO diet for 10 weeks.^26,44^ Participants with normoglycemia lost 0.43 kg more on the high-CHO diet (*P* = 0.03), and there was no difference in weight loss among participants with prediabetes assigned to either high- or low-CHO diets. Participants with T2D tended to lose more weight on the low-CHO diet than their counterparts on the low-fat diet, although the difference was not significant (−2.04 kg; *P* = 0.07). The investigators of the NUGENOB study found that those with low FI lost 0.42 kg more on the low-fat/high-CHO diet (*P* = 0.046) versus high-fat/low-CHO diet, and no difference in weight loss was observed for those with high FI (*P* = 0.84) or between low- and high-FI groups (*P* = 0.33).^26^

#### 3.1.4 | Monounsaturated Fatty Acids in Obesity (MUFObes) study

The MUFObes study randomized 131 participants to one of three ad libitum diets for 6 months after achieving at least 8% weight loss^45^ to investigate the impact of these diets on WLM. The diets under investigation included the following: (1) MUFA, a high-fiber (>30 g/10 MJ), high-energy density, moderate-fat diet (35%–45% energy from fat) with >20% of fat coming from MUFAs; (2) Nordic Nutrition Recommendations (NNR), high-fiber (>30 g/10 MJ), low-energy density, low-fat diet (20%–30% energy from fat); and (3) Control, lower in fiber (<30 g/10 MJ), high in saturated fatty acids (>15% of energy), and high in energy density. Alcohol (<5% of energy) and protein (10%–20% of energy) were similar across the diets. In a secondary analysis, 104 participants were stratified by FPG and FI after the initial 8-week weight loss phase. The cutoff for high FPG was lowered to 5.0 mmol/L because no pre-weight loss glucose values were available and FPG is often reduced following weight loss. During the 6-month weight maintenance period, participants with FPG > 5.0 mmol/L gained 2.73 kg on the MUFA diet and 4.16 kg on the Control diet compared with −0.05-kg weight change on the NNR diet.^27^ The cutoff for high FI was based on the median post-weight loss value (50 pmol/L). FI alone was not found to be a significant predictor of weight change across the three diets. However, a significant difference in weight change was observed in participants with high FPG and high FI between the NNR diet (−0.05 kg) and Control diet (6.91 kg).

#### 3.1.5 | Hjorth et al.: Retrospective analysis of NCT00143936

A 2-year randomized clinical trial in 307 adults with obesity compared achieved weight loss between individuals assigned to consume either a low-fat diet or a low-CHO diet following recommendations from the book, *Dr. Atkins’ New Diet Revolution*.^46^ Both diets were consumed ad libitum, and intensive behavioral counseling was provided to participants in both diet conditions. Approximately 11% weight loss was achieved by month 6, and approximately 7% weight loss was maintained by the 2-year assessment with no difference in achieved weight loss between the low-fat and low-CHO diets.^46^ In a retrospective analysis by Hjorth et al., participants with prediabetes and high FI lost 7.2 kg more weight (*P* = 0.005) on a low-fat versus low-CHO diet, whereas participants with prediabetes and low FI lost 6.2 kg more weight on the low-CHO diet (*P* = 0.088). There was no difference in weight loss between diets for those with normoglycemia and high or low FI (*P*≥ 0.16).^28^

#### 3.1.6 | POUNDS LOST study

The POUNDS LOST study represents one of the most influential dietary weight loss studies conducted to date. Participants (*n* = 811) were randomly assigned to one of four experimental diets varying in macronutrient composition. The primary analysis indicated no significant difference in weight loss after 2 years among the four diets and noted that attendance to the group sessions was the strongest predictor of weight loss regardless of the macronutrient content of the diet.^47^ A secondary analysis of the POUNDS LOST study investigated whether pretreatment FPG, FI, and insulin resistance (HOMA-IR) affected weight loss on the experimental diets.^29^ The role of dietary fiber was also investigated. Findings of this secondary analysis suggested that people with normal glycemia (FPG < 100 mg/dL) lost the most weight on a low-fat/high-protein diet (55% of energy from CHO), whereas subjects with insulin resistance lost the most weight on a high-fat/high-protein diet (35% of energy from CHO). In addition, the authors concluded that people with prediabetes (FPG ≥ 100 mg/dL) and FI below the median lost the most weight on diets that included at least 35 g of fiber daily.^29^

#### 3.1.7 | A to Z study

The A to Z study compared popular, named diets that span a spectrum of CHO intakes using a 12-month randomized clinical trial with women in premenopause. Participants were assigned to one of the following diets: Atkins (very-low-CHO), Zone (40% of energy from CHO), LEARN (55%–60% of energy from CHO), or Ornish (<10% of energy from fat, highest dietary CHO). The principal finding from the original trial was that women assigned to the Atkins diet group had the greatest weight loss at 12 months.^48^ A secondary analysis of the A to Z study compared weight loss in the Atkins and Ornish diets—representing the greatest difference in CHO intake—by insulin resistance status.^30^ Insulin resistance was determined by tertiles of FI concentrations, and those in the lowest tertile (<6.9 μIU/mL) were defined as “insulin sensitive” and those in the highest tertile (>10.6 μIU/ml) were defined as “insulin resistant.” The insulin resistance status by diet interaction showed that insulin-resistant women consuming the low-fat Ornish diet were less adherent to the diet prescription and lost less weight compared with those consuming the low-CHO Atkins diet. On the other hand, adherence and weight loss on the Atkins diet were not affected by insulin resistance status.^30^

### 3.2 | Prospective trials

#### 3.2.1 | Shyam et al. 2013

Seventy-seven young Asian women with a history of gestational diabetes mellitus were randomized to follow either conventional healthy dietary recommendations (CHDR) or CHDR plus additional advice to reduce the glycemic index of their diet.^31^ The primary outcome for this study was 2-h glucose concentration during a 75-g OGTT, but prespecified analyses of changes in body weight between diets and by glycemic and insulinemic status were noted in the primary manuscript. The authors reported that body weight, body mass index (BMI), and waist circumference were reduced at month 6 compared with baseline in the low glycemic index diet (within-group comparisons), but only waist circumference was reduced in the CHDR group. However, no between-group statistical comparisons are reported and overall anthropometric changes were modest and not clinically meaningful. Baseline glucose status did not significantly affect changes in anthropometric outcomes. Among participants with high FI (≥2 μIU/mL), the authors report that percentage weight loss was quantitatively greater when consuming the low glycemic index diet (−3.1% vs. + 0.2%) but the difference was not statistically significant (*P* = 0.09) and, as noted previously, did not meet the widely accepted threshold of ≥5% weight loss for clinical significance.^31^

#### 3.2.2 | Ballesteros-Pomar et al. 2009 (16-week weight loss data) and Fernández et al. 2012 (1-year weight loss maintenance data)

Results from this small prospective study were published in two manuscripts reporting 16-week weight loss outcomes^32^ and WLM outcomes at week 52.^33^ At baseline, participants were classified as either insulin sensitive (*n* = 15) or insulin resistant (*n* = 21) based on the results of an OGTT and calculation of HOMA-IR. Participants were then randomly assigned to either a reduced-CHO diet (40% of energy from CHO, 30% protein, and 30% fat) or high-CHO diet (55% CHO, 15% protein, and 30% fat). Most studies included in this review modified CHO content by altering dietary fat (i.e., reduced-CHO, high-fat vs. high-CHO, low-fat) and keeping dietary protein similar between diets; however, both prescribed experimental diets in this study had 30% of energy from fat, and dietary protein was modified to offset changes in dietary CHO. Because of the small sample size and stratification by insulin sensitivity status, the number of participants in each condition is very small (sensitive + reduced-CHO: *n* = 8, sensitive + high-CHO: *n* = 7, resistant + reduced-CHO: *n* = 10, resistant + high-CHO: *n* = 11) and likely underpowered to statistically detect a glucose metabolism phenotype by diet interaction. The authors reported that the sample size was expected to be sufficient to detect a3–4 kg difference in weight loss, which is a large difference. Further, this power calculation was based on findings from studies that used a full food provision protocol through General Clinical Research Centers that likely reduced variability in dietary adherence and achieved weight loss compared with their protocol that used dietitian-led counseling sessions to promote adherence to the prescribed diets. Given those caveats, the authors reported that weight loss^32^ and WLM^33^ were not different between diets nor were affected by insulin sensitivity status.

#### 3.2.3 | Rock et al. 2016

Women with overweight or obesity but without diabetes (*n* = 245) were classified as insulin sensitive or insulin resistant based on HOMA-IR and then randomized to one of three experimental diets for 12 months: low-fat (20% of energy from fat, 65% CHO), reduced-CHO (45% CHO, 35% fat), or reduced-CHO + walnut-rich (45% CHO, 35% fat, 18% walnuts). The overall time × diet × insulin resistance was not significant, and no differences in weight loss among the diets were observed for insulin-resistant women. However, the authors did report that insulin-sensitive women tended to lose more weight on the low-fat diet (7.5 kg, *P* = 0.06) and reduced-CHO + walnut diet (8.1 kg, *P* = 0.04) compared with the reduced-CHO diet (4.3 kg).^34^

#### 3.2.4 | Cornier et al. 2005

Cornier et al. conducted a small, randomized weight loss trial of women with obesity and normoglycemia to test whether achieved weight loss depended on both insulin sensitivity and macronutrient composition.^35^ Insulin-sensitive (*n* = 12) and insulin-resistant (*n* = 9) women were enrolled into the study based on FI concentrations of <10 and >15 μU/mL, respectively. All foods were prepared in a metabolic research kitchen and provided to participants to consume at home for 16 weeks. The diets were designed to achieve a 400-kcal daily energy deficit. The authors hypothesized that insulin-sensitive women would lose more weight on a high-CHO (60% of energy)/low-fat (20%) diet compared with a low-CHO (40%)/high-fat (40%) diet. Conversely, they hypothesized that insulin-resistant women would lose more weight on the low-CHO/high-fat diet. Results from this small, but well-controlled study were consistent with the authors’ a priori hypotheses. Women with FI < 10 μU/mL (i.e., insulin sensitive) lost 13.5 ± 1.2% of their body weight on the high-CHO, low-fat diet compared with 6.8 ± 1.2% on the low-CHO, high-fat (*P* < 0.002 between groups). In women with FI > 15 μU/mL (i.e., insulin resistant), weight loss was greater with the low-CHO, high-fat diet (13.4 ± 1.3%) versus the high-CHO, low-fat diet (8.5 ± 1.4%; *P* < 0.04 between groups).^35^

#### 3.2.5 | Comprehensive Assessment of Long-term Effects of Reducing Intake on Energy (CALERIE)

In a pilot study conducted as part of the CALERIE trial, Pittas et al. examined if baseline INS-30 or HOMA-IR predicted weight change on high versus low glycemic load diets. Thirty-four individuals with a BMI between 25.0 and 29.9 kg/m^2^ and normal FPG (<100 mg/dL) were randomized to either a calorie-restricted high glycemic load or low glycemic load diet for 6 months.^36^ All foods were provided to participants, and they received regular group meetings and individual sessions with a registered dietitian to encourage adherence to the prescribed diets. Those with high INS-30 lost more weight on the low glycemic load diet (−10 kg vs. −6 kg; *P* = 0.027), whereas those with low INS-30 lost quantitatively more weight on the high glycemic load diet, but the difference was not statistically significant (−8 kg vs. −6 kg; *P*= 0.25).^36^

#### 3.2.6 | Ebbeling et al. 2007

In an 18-month randomized weight loss trial, 73 adults aged 18–35 were randomized to consume either a low-GL diet (40% of total energy from CHO and 35% from fat) or low-fat diet (55% CHO and 20% fat). Blood samples for determining INS-30 were collected at baseline and frozen until the time of analysis, which was after the intervention had been completed. Overall, weight loss did not differ between the low GL and low-fat diets, but INS-30 was identified as a significant effect modifier. For those with INS-30 values above the median, weight loss at 18 months was greater on the low GL diet (−5.8 kg) compared with the low-fat diet (−1.2 kg; *P* = 0.004). Weight loss did not differ between diets among participants with baseline INS-30 values below the median.^37^

#### 3.2.7 | Gardner et al. Pilot Trial and Diet Intervention Examining The Factors Interacting with Treatment Success (DIETFITS) trial

In a randomized pilot trial of 61 adults without T2D, participants followed either a low-fat or low-CHO diet for 6 months.^38^ Despite substantial weight loss of 9.0 ± 6.5 kg, no main effects of diet or insulinemic status or insulinemic status by diet-type interaction on weight loss were detected.^38^ Subsequently, the authors conducted the DIETFITS trial, which represents the most robust randomized experiment to determine whether pretreatment insulin secretion affects weight loss outcomes in low-CHO and low-fat diets.^39^ Interactive effects of genotype or baseline insulinemic status by diet type were examined in 609 adults with overweight or obesity. After 12 months, weight change was not different between groups (−5.3 kg vs. −6.0 kg for low-fat and low-CHO diets, respectively), and no diet-genotype (*P* = 0.20) or diet-insulin secretion interactions (*P* = 0.47) were observed.

Subsequent secondary analyses of the DIETFITS data found that both experiment diets decreased glycemic load relative to usual intakes^19^ and resulted in differences in dietary fiber intakes that were lower than expected between diets.^49^ With data from both diet groups combined, measures related to CHO intake (i.e., glycemic load/index and added sugar) were consistent and strong predictors of weight loss, whereas the change in total fat intake was not associated with weight loss. An effect mediation analysis also showed that the effect of calorie reduction on observed weight loss was largely mediated by a reduction in glycemic load. The authors—notable proponents of the Carbohydrate-Insulin Model—interpreted these findings as being broadly supportive of said model among all participants, regardless of insulin secretion status. Additionally, participants in the highest quintile of both INS-30 and glycemic load reduction lost more weight than any other quintile at every time point (*P*-value for interaction: 0.0009 at month 3, 0.01 at month 6, and 0.07 at month 12) suggesting potential effect medication by insulin secretion on the relationship between glycemic load reduction and weight loss.^19^

Still, another secondary analysis of the DIETFITS data sought to investigate the contributions of dietary adherence, diet quality, caloric restriction, and proteomic and gut microbiota signatures to short-term (6 months) and long-term (12 months) weight loss.^50^ Overall, adherence to the assigned diet was strongly associated with weight loss within each group and performed better than overall caloric restriction as a predictor of weight loss. The receiver operating characteristic (ROC) analysis showed an area under ROC (AUROC) = 0.58 for calorie restriction in the low-CHO group compared with an AUROC = 0.73 for percentage of calories from CHO. Similarly, in the low-fat group, the AUROC for calorie restriction was 0.51 versus 0.67 for percentage of calories from fat. In pursuing the possibility of deeply personalized diet prescriptions, the authors suggested that individuals with a habitual diet that is relatively low in dietary fat and present with an expectedly high respiratory quotient (RQ; indicating high-CHO oxidation) are likely ideal candidates for weight loss using a low-CHO/higher fat strategy. A greater reduction in RQ was associated with greater weight loss (*r* = 0.22, *P* = 0.001) among individuals in the low-CHO diet group, and this pattern of RQ reduction with a low-CHO diet represents the expected metabolic response to the prescribed dietary pattern. Interestingly, participants who did not experience the expected reduction in RQ with the low-CHO diet also had higher INS-30. This unexpected metabolic response to the low-CHO diet was not explained by differences in overall calorie reduction or dietary adherence. Nonetheless, this somewhat contradictory finding suggests that people with insulin resistance (i.e., high INS-30) consuming a diet that is habitually low in fat may not benefit as much as expected when consuming an energy-restricted low-CHO diet.

## 4 | SUMMARY AND OPPORTUNITIES FOR FUTURE RESEARCH

Conceptually, it seems reasonable and logical that modifications to dietary CHO (i.e., *externally derived* CHO) would benefit people with an impaired ability to manage their *internal* CHO status (i.e., prediabetes, T2D, and insulin resistance). In practice, the interaction(s) of pretreatment glycemic and/or insulinemic status with dietary CHO for weight loss are exceptionally complex, and different approaches to define pretreatment status and multiple strategies for modifying CHO intake have resulted in variable findings as presented above. The overall intervention design also likely plays an important role in determining treatment response. For example, adherence to the dietary protocol consistently ranks as the strongest predictor of weight loss success,^47^ including in investigations of low-fat versus low-CHO diets.^50^ Study designs that intentionally counteract the impact of dietary adherence (i.e., controlled feeding trials) provide critical evidence on the impact of diet composition on weight loss under idealized conditions where intake is closely monitored and controlled. However, controlled feeding trials provide little information as to the performance of dietary strategies in the real world where foods are freely chosen and consumed at will. On the other end of the spectrum, more pragmatic and/or counseling-based interventions are useful for determining whether *recommendations* to follow a particular dietary pattern affect achieved weight loss, but self-reported assessments of dietary intake are notoriously problematic,^51^ which makes evaluations of self-reported dietary intake and adherence nearly impossible to determine accurately and reliably.

Despite these considerations, six of seven retrospective and three of eight prospective studies identified at least one significant glycemic/insulinemic status by diet interaction favoring a CHO-modified diet for people with impaired glucose metabolism. It should be noted that most of the prospective studies included <100 participants and were potentially limited in statistical power to detect the glucose metabolism phenotype by diet type interactions.^31–33,35–38^ Among people with normal glucose metabolism, it is more common that weight loss is either not different or tends to be greater with CHO-modified diets compared with high-CHO, low-fat diets.^24–26,28^ Notably, no studies were identified wherein a CHO-modified diet resulted in less weight loss versus a low-fat diet in people with impaired glucose metabolism. Based on these collective findings, it is reasonable to conclude that reduced-CHO dietary patterns with a low glycemic index/load and high-fiber content should be the first-line dietary approach for those with impaired glucose metabolism. Those with normal glucose metabolism could likely do equally or nearly equally well on a CHO-modified or low-fat diet. However, this conclusion should not be misconstrued as a definitive endorsement of CHO-modified diets for weight loss in people with impaired glucose metabolism. Rather, this conclusion represents a suggestion based on currently available evidence, which is not particularly strong nor convincing. The following sections of this review will consider limitations of the extant data and possible approaches for improving the quality and rigor of future research to inform more robust dietary recommendations based on pretreatment—and possibly early in-treatment—factors.

### 4.1 | General limitations of the evidence

First, the limitations of the current review and uniqueness of the search strategy employed for identifying relevant publications should be addressed. A vast literature exists describing the effects of weight loss on *changes in glycemic and insulinemic status*. Of course, the goal of the search for this review was to identify publications addressing a question in the opposite direction, that is, the *effect of glycemic and insulinemic status* on weight loss. A simple search on PubMed using the terms [(“glucose” OR “insulin”) AND “weight loss”] with filters applied for clinical trial, randomized controlled trial, meta-analysis, and systematic review yielded 3774 results as of March 16, 2023. Because this search strategy does not specify the directionality of the relationship between glucose or insulin and weight loss, examples of both study types are included in the search results. A university librarian with expertise in search strategies for scoping reviews, systematic reviews, and meta-analyses was consulted for this search strategy and suggested the use of additional terms such as “baseline” and “pretreatment.” One example of a much more limited search strategy that was used for the literature search included the terms [(“glucose” OR “insulin”) AND “weight loss” AND (“baseline” OR “pretreatment”) AND “carbohydrates”] with filters applied for clinical trial, randomized controlled trial, meta-analysis, and systematic review yielded only 100 results as of March 16, 2023. This limited the number of publications to be reviewed for inclusion, but likely at the risk of excluding potentially relevant publications. A spectrum of search strategies and terms between these two extremes were used to identify relevant publications for this review, as were manual searches of the reference lists on identified publications.

The limitations of this search strategy raise a very plausible circumstance of missed publications that would have been eligible for inclusion in this review. Although this limitation is acknowledged, given that no studies were identified that favored a low-fat diet for people with impaired glucose metabolism, it is unlikely that a sufficient number of such publications would have been missed in order to justify reversing the conclusion that CHO-modified diets can be recommended as a first-line behavioral weight loss strategy for this population. Even if additional publications were identified that demonstrated no difference in weight loss by diet type, the recommendation to initiate behavioral weight loss with a CHO-modified diet would not be expected to cause harm. In such a hypothetical case, the likelihood of successful weight loss would not be expected to differ by the initial diet prescription, and the selection of the initial dietary pattern could likely be determined by random allocation, practitioner recommendation, or patient choice with little to no difference in success among these selection strategies. This assertion is supported by randomized experiments designed to assess the impact of allowing participants to choose their preferred dietary pattern. In one such randomized clinical trial of 207 patients at a Veterans Affairs medical center with a BMI of at least 30 kg/m^2^, participants were either assigned a low-CHO or low-fat diet by random allocation or allowed to choose between the diets.^52,53^ Participants in the choice condition were given a second opportunity to switch diets after 12 weeks. The first important observation is that participants in the choice condition were likely to choose a dietary pattern that most closely aligned with their habitual diet,^52^ and only five of 105 participants elected to switch their diet assignment at week 12.^53^ Ultimately, the allowance of patient choice had no meaningful impact on weight loss after the 48-week interventions (mean difference: −1.1 kg, 95% CI: −2.9–0.8 kg, *P* = 0.26).^53^

Next, the limitations of the existing literature in support of recommending a CHO-modified diet for people with impaired glucose metabolism will be considered. The finding that six of seven retrospective studies but only three of eight prospective studies support a phenotype by diet interaction needs to be interpreted carefully. First, findings from the vast majority of retrospective analyses agree with meta-analytical findings that generally support a modest weight loss benefit for low-CHO versus low-fat diets.^22–25^ Considering the physiologic plausibility of the Carbohydrate-Insulin Model and known effects on insulin action on fat metabolism, it is not surprising that the potential benefit of a CHO-modified diet would be observed and accentuated in people with impaired glucose metabolism. However, the number of investigations that have specifically addressed this potential phenotype by diet interaction is comparatively limited. There have been numerous low-CHO versus low-fat comparisons spanning the spectrum from normal glucose metabolism to overt T2D, but the majority of studies have utilized inclusion/exclusion criteria that specifically target a population of interest, which limits the ability to investigate the moderating effect of glucose and/or insulin status within defined study cohorts. It is also worth noting that six of seven retrospective studies (five of which showed a benefit of CHO-modified diets) identified by the current review were conducted by the same research group^26–29^ using a consistent analytic approach and methods for defining participants with impaired glucose metabolism. Additionally, three authors of this review (HRW, JOH, and RDS) are co-authors on one of these retrospective analyses.^28^ With the exceptions of the DIETFITS trial^39^ and the study by Rock et al.,^34^ prospective randomized clinical trials with prespecified analyses of a phenotype by diet interaction are mostly limited to small (i.e., possibly underpowered) clinical trials with <100 participants. Notably, neither of these two larger prospective trials found any weight loss benefit of the CHO-modified diets compared with high-CHO diets.

The DIETFITS trial in particular was a sufficiently large and well-powered study designed to test the hypothesis that participants with insulin resistance (i.e., high INS-30) would lose more weight on a “healthy” low-CHO diet compared with a “healthy” low-fat diet. Further analyses of the diet prescriptions in DIETFITS warrant the emphasis on the “healthy” descriptor of the two diets. Both diets used a general strategy of reducing intakes of added sugars and refined grain products to produce “heathy” dietary patterns, which had the additional effects of reducing the glycemic load of both diets relative to the participants’ habitual diets^19^ and creating a lower than anticipated difference in fiber intake between the diet groups.^49^ The most definitive study conducted to date refuting the superiority of a low-CHO diet for weight loss in people with insulin resistance appears to be somewhat less definitive in light of these secondary analyses. However, because reported overall CHO intake was markedly reduced in the low-CHO diet versus low-fat diet, future prospective studies in this area might focus on designing study diets that are sufficiently distinct in glycemic load, CHO quality, and fiber content rather than focusing specifically on the overall amount of dietary CHO.

### 4.2 | Why suggest a “more of the same” approach to behavioral weight loss?

Readers of this review might question the recommendation to prescribe a reduced-CHO dietary pattern—especially those with a low glycemic index/load and high-fiber content—for weight loss in people with impaired glucose metabolism due to the pervasive nature of such diets in both the scientific and popular literature/media. Although a low-fat (and thus high-CHO) dietary pattern gained considerable popularity in the 1980s and 1990s, dietary strategies to reduce or modify CHO intakes have dominated the popular diet landscape for the past several years.^15^ This observation raises a very salient and logical question: If CHO-modified diets are beneficial and such a large portion of the US adult population has impaired glucose metabolism,^54^ why has their considerable popularity and use by the general public not resulted in a measurable public health impact on obesity? First, as has been noted, the evidence in support of this recommendation is generally weak, and decisions on dietary choices or prescriptions should consider other contextual factors affecting eating behaviors and preferences. Second, it may be that CHO-modified diets are superior for weight loss *when* they are consumed, but that poor adherence to the dietary pattern undermines its potential public health impact. Put another way, the problem with behavioral weight loss may not be the diet prescription per se, but rather the inability of most people to sufficiently adhere to the prescription. More and rigorous behavioral science research could address the issue of poor uptake and maintenance of health-related behaviors, including the consumption of CHO-modified diets for weight loss. Behavioral researchers and clinicians must also move beyond viewing adherence as resulting from merely a lack of willpower and acknowledge the diverse and interacting factors affecting eating behaviors. Such behavioral research should be rooted in theoretically-based interventions and described behavioral change techniques.^55,56^ The National Institutes of Health (NIH) Stage Model for Behavioral Intervention Development provides a useful framework for the creation, modification, and/or adaptation of behavioral interventions that are both more potent and ultimately implementable.^57^ The consistent application of these rigorous behavioral research methods and focus on mechanisms of behavior change could lead to more effective and personalized behavioral interventions that deliver the most effective behavior change techniques to the right person, at the right time, and in the right context (i.e., Precision Behavioral Medicine).

### 4.3 | Innovative and rigorous experimental designs to optimize and tailor dietary strategies

This section describes some potential opportunities for future research to confirm or refute the recommendation to prescribe CHO-modified diets for weight loss to people with impaired glucose metabolism. First and foremost, there is an urgent need for additional and sufficiently large preregistered randomized trials that include direct tests of phenotype (e.g., insulin sensitive vs. insulin resistant) by diet (e.g., low-CHO vs. low-fat) interactions. Other potentially fruitful areas of research include studies on the interaction of dietary patterns with exercise and whether the optimum macronutrient distribution is different between initial weight loss and long-term weight loss maintenance. In these circumstances, co-engagement in an exercise program and early weight loss would both be expected to improve insulin sensitivity and glucose metabolism. Whether dietary strategies should be altered during the course of obesity treatment based on intervention-derived improvements in metabolic status is largely unexplored. Previous research has also demonstrated considerable intraindividual and interindividual variability in glycemic and insulinemic responses to food. For example, among 63 healthy men and women representing a range of ages and BMI, the mean glycemic index of white bread was 62 ± 15, but the intraindividual and interindividual coefficients of variation were 20% and 25%, respectively.^58^ Researchers designing, conducting, and reporting results from these trials must consider how alternative methods for identifying participants with impaired glucose metabolism (e.g., fasting vs. postprandial measures and glucose vs. insulin vs. both), methods for modifying CHO intake (e.g., reduced overall CHO intake, reduced glycemic index/load, and higher fiber), and experimental design (e.g., counseling-based vs. controlled-feeding) affect the interpretation of their work within the broader literature.

Although observational studies and retrospective analyses of past randomized trials can generate new hypotheses and inform experimental approaches, prospective randomized experiments should be the foundation for future research in this area. However, the nature of the randomized trials could vary considerably in size, scope, and cost. For example, the NIH-sponsored Nutrition for Precision Health consortium^59^ will test three dietary patterns under highly controlled settings to metabolically phenotype individuals and their physiological responses to defined foods and dietary patterns. Although these studies will be highly controlled and limit the influence of dietary adherence on observed responses, individual contextual factors such as perceived appetite, psychosocial factors, and social determinants of health will also be collected. Collectively, these intensive physiological and contextual data may help to inform future studies investigating how likely people with an identifiable phenotypic and contextual profile are to adopt and maintain a particular dietary pattern. On the other end of the continuum, registry-based randomized trials have been suggested as an innovative and low-cost method for rigorously assessing the effectiveness of low-intensity lifestyle-based interventions in large numbers of people with reliable outcome measures derived from the parent registry.^60,61^ Although experimental approaches at both ends and all along this continuum can contribute to the question of whether CHO-modified are superior for weight loss in people with impaired glucose metabolism, the use of randomized experiments is a common and essential component of suggested future research possessing the highest probative value.

The Multiphase Optimization Strategy (MOST) Framework represents a particularly attractive approach for developing and optimizing lifestyle-based interventions.^62,63^ An essential component of MOST is a concept of intervention optimization referred to as Intervention EASE: developing the most effective intervention package possible within key constraints of affordability, scalability, and efficiency. This approach is consistent with the aforementioned NIH Stage Model, which emphasizes considerations of both the potency and implementability of the intervention package even at the earliest stages of intervention development. Although there is no specific MOST “design,” experimental approaches under the MOST Framework typically utilize a type of randomized factorial experiment (i.e., full, fractional, or conditional) and a phased approach (preparation, optimization, and evaluation) to determine the usefulness of distinguishable intervention components and their interactions within a multicomponent intervention package. Factorial designs could be used to test the main effects and interactions of distinct CHO-modification techniques (e.g., reduced overall CHO, reduced glycemic index of CHO-containing foods, and higher fiber content) or investigate how a particular CHO-modified diet performs relative to and in combination with other lifestyle-based interventions such as exercise, sleep, or time-restricted eating.

The Sequential Multiple Assignment Randomized Trial (SMART) is a specific experimental approach under the MOST Framework that seeks to develop personalized adaptive treatment sequences based on pretreatment factors and early in-treatment responses to initial interventions. In a SMART, participants not achieving a predefined benchmark for success to initial randomized interventions are re-randomized to second-stage interventions.^64^ The nature of second-stage interventions can vary considerably but generally represent an intensification of the intervention approach such as augmenting initial interventions (e.g., augmenting dietary counseling with provided “on-plan” meals) or adding new intervention components (e.g., addition of time-restricted eating or exercise training). An inherent aim of SMARTs is to collect a large amount of contextual information from participants prior to starting the intervention (e.g., biological, sociodemographic, psychosocial, environmental, and behavioral) and during the early phases of the intervention (e.g., initial success, consistency of behaviors, and adherence) to ultimately predict which intervention sequence is most likely to be successful for an individual given these unique factors. These pretreatment and early in-treatment factors are referred to as baseline and intermediate tailoring variables, respectively. Examples of potential uses for a SMART in the context of CHO-modified diets include the consideration of pretreatment glycemic/insulinemic status as a baseline tailoring variable, achieved weight loss or a measure of dietary adherence as intermediate tailoring variables, and second-stage interventions that either intensify the provision of CHO-modification strategies or combine them with other lifestyle interventions.

## 5 | CONCLUSION

As identified in this review, the best available evidence is relatively weak but suggests a possible benefit (and very unlikely harm) in using CHO-modified diets as a first-line dietary option for behavioral weight loss, especially in those with impaired glucose metabolism. Because of the weak nature of the recommendation and research showing that weight loss can be achieved via a variety of approaches, clinicians should consider this recommendation in the context of their patients’ habitual diets, preferences, and other contextual factors (e.g., cost/resources, family dynamics/preferences, and availability) that may affect their ability to adhere to a particular dietary pattern. There remains a critical need for additional and much more rigorous behavioral, physiological, and socioecological research vis-à-vis personalizing dietary recommendations for weight loss.

## Figures and Tables

**TABLE 1:** Characteristics of normal glucose tolerance, impaired fasting glucose, impaired glucose tolerance, and combined impaired fasting glucose and glucose tolerance during an OGTT.

Glucose tolerance status	Fasting glucose concentration	2-h glucose concentration	First-phase insulin response^1^	Early-phase insulin response^2^	Late-phase insulin response^3^
Normal glucose tolerance	<5.5 mmol/L	<7.8 mmol/L	Normal/Reference	Normal/Reference	Normal/Reference
Impaired fasting glucose	5.6–7.0 mmol/L	<7.8 mmol/L	↓	↓	↔
Impaired glucose tolerance	<5.5 mmol/L	7.8–11.0 mmol/L	↓	↓	↓↓↓
Combined impairment	5.6–7.0 mmol/L	7.8–11.0 mmol/L	↓	↓	↓↓↓

1 Insulin secretory response during the initial 10 min of an OGTT.

2 Insulin secretory response during the initial 30 min of an OGTT.

3 Insulin secretory response during minutes 60–120 of an OGTT.

**TABLE 2:** Summary of included retrospective studies.

Study name (reference)	CHO modification strategy (ies)	Glycemic/insulinemic indicator(s)	Benefit of CHO-modified diet in participants with impaired glucose metabolism
DiOGenes Study^26^	Low glycemic load	Fasting plasma glucose, fasting insulin	Less weight regain on low glycemic load diet in participants with prediabetes, low fasting insulin, and prediabetes + low fasting insulin
SHOPUS Trial^26^	High-fiber	Fasting plasma glucose, fasting insulin	Greater weight loss on high-fiber diet in participants with prediabetes, low fasting insulin, and prediabetes + low fasting insulin
NUGENOB Study^26^	Reduced-CHO diet	Prediabetes or type 2 diabetes	No
MUFObes Study^27^	Nordic Nutrition Recommendations Diet (NNR; high-fiber, low energy density, low-fat)	Fasting plasma glucose, fasting insulin	Less weight regain on NNR Diet in participants with high fasting plasma glucose and high fasting plasma glucose + high fasting insulin
Hjorth Secondary Analysis of NCT00143936^28^	Atkins Diet (very-low-CHO)	Prediabetes and fasting insulin	Participants with prediabetes + low fasting insulin lost more weight on the Atkins Diet, but those with prediabetes plus high fasting insulin lost more on the low-fat diet.
POUNDS LOST Study^29^	Reduced-CHO+ high-protein and high-fiber	Fasting plasma glucose and HOMA-IR	Participants with prediabetes lost the most weight on the reduced-CHO+ high-protein diet, insulin-resistant participants lost more weight on diets with >35 g of daily fiber.
A to Z Study^30^	Atkins Diet (very-low-CHO)	Fasting insulin	Weight loss was greatest on the Atkins Diet regardless of fasting insulin. Participants with high FI had the poorest adherence and lost less weight on the low-fat Ornish Diet.

Abbreviations: CHO, carbohydrate; FI, fasting insulin; HOMA-IR, homeostatic model assessment for insulin resistance; NNR, Nordic Nutrition Recommendation.

**TABLE 3:** Summary of included prospective studies.

Study name (reference)	CHO modification strategy (ies)	Glycemic/Insulinemic indicator(s)	Benefit of CHO-modified diet in participants with impaired glucose metabolism
Shyam et al. 2013^31^	Reduced glycemic index	Fasting and 2-h post-OGTT glucose and insulin	No
Ballesteros-Pomar et al. 2010^32^ and Fernandez et al. 2012^33^	Reduced-CHO + high-protein	HOMA-IR and peak insulin concentration during an OGTT	No
Rock et al. 2016^34^	Reduced-CHO diet, reduced-CHO diet enriched with walnuts	HOMA-IR	No
Cornier et al. 2005^35^	Reduced-CHO	FI	Participants with high fasting insulin lost more weight on the reduced-CHO diet.
CALERIE Pilot^36^	Low glycemic load	INS-30	Participants with high INS-30 lost more weight on the low glycemic load diet.
Ebbeling et al. 2007^37^	Low glycemic load	INS-30	Participants with high INS-30 lost more weight on the low glycemic load diet.
Gardner et al. 2016 Pilot^38^	Reduced-CHO	INS-30	No
DIETFITS^39^	Reduced-CHO	INS-30 and predictive genotype	No

Abbreviations: CHO, carbohydrate; HOMA-IR, homeostatic model assessment for insulin resistance; INS-30, insulin concentration 30 min into an OGTT; OGTT, oral glucose tolerance test.

## Data Availability

The authors have no data to share for this manuscript.

## Abbreviations:

AUROC: area under receiver operating characteristic
BMI: body mass index
CHDR: conventional healthy dietary recommendations
CHO: carbohydrate
DIETFITS: Diet Intervention Examining the Factors Interacting with Treatment Success
DiOGenes: Diet, Obesity, and Genes
FI: fasting insulin
FPG: fasting plasma glucose
HOMA-IR: homeostatic model assessment for insulin resistance
IFG: impaired fasting glucose
IGT: impaired glucose tolerance
INS-30: insulin concentration 30 min into an OGTT
MOST: Multiphase Optimization Strategy
MUFObes: Monounsaturated Fatty Acids in Obesity
NGT: normal glucose tolerance
NIH: National Institutes of Health
NNR: Nordic Nutrition Recommendations
NUGENOB: Nutrient-Gene Interactions in Human Obesity
OGTT: oral glucose tolerance test
ROC: receiver operating characteristic
SHOPUS: Supermarket Intervention
SMART: Sequential Multiple Assignment Randomized Trial
T2D: Type 2 diabetes
WLM: weight loss maintenance
